# UWB Low-Profile Boat-Radiator Antenna (BRA) with Dual C-Shape Co-Radiative Ground for Multi-Standard Communication Networks

**DOI:** 10.3390/s20247051

**Published:** 2020-12-09

**Authors:** Chungang Zhang, Yongjun Xie, Legen Dai

**Affiliations:** School of Electric and Information Engineering, Beihang University, Beijing 100191, China; yjxie@buaa.edu.cn (Y.X.); dlg9105@buaa.edu.cn (L.D.)

**Keywords:** UWB antenna, low profile, uniform radiation pattern, multi-standard communication network

## Abstract

Multiple standard communication networks operate in the frequency band of 1.8–6 GHz, which makes lots of antennas available in the limited space. To solve the problem of interference and improve the performance of these antennas, an ultra-wideband (UWB) antenna is presented. It consists of a boat-radiator and a dual C-shape co-radiative ground (DCCRG). One half of the DCCRG plays a role of the ground of a co-planar waveguide fed to the proposed boat-radiator antenna (BRA), while the other half works as a multiple order L-resonant circuit to broaden the lower operating band. Uniform bidirectional radiation is presented with the size of 0.25 λ × 0.375 λ × 0.0063 λ over the frequency band of 1.7–6.3 GHz (115%). The proposed antenna achieves around twice the bandwidth (60%) of the same structured antenna without the structure acting as a multiple order L-resonant circuit. Moreover, the stable boresight gain of 3.6 dBi ± 1.25 dBi is realized over the operating band.

## 1. Introduction

Over the last few decades, mobile communication technology has experienced explosive prosperity and tremendously facilitated people’s lives around the globe [1]. The third generation (3G) communication networks (2.1 GHz), the fourth generation (4G) communication network (1.8 GHz, 2.3 GHz, 2.5 GHz in China), Bluetooth (2.45 GHz), and WIFI (2.4 GHz, 5 GHz) have been widely used. Moreover, the fifth generation (5G) communication system will meet the increasing demands of higher data rates, larger capacity, and lower latency in the future, which is expected to promote the prosperity of the Internet of Things [2,3]. The prosperity of 5G will bring more antennas employed in the frequency band below 6 GHz (3.6 GHz, 4.8 GHz in China). Therefore, multiple standard communication networks operate in the frequency band of 1.8–6 GHz. Moreover, these communications networks will exist at the same time for a period, which makes the phenomenon of numerous antennae operating for different communication networks serious.

The coexistence of multi-standard communication networks has resulted in a large number of antennas working in a limited space, which will generate severe interference among the antennas and reduce their performance. The complexity of the design, fabrication, and installation of the antennas will increase as well. To solve such problem, ultra-wideband (UWB) antennas have been developed in recent years.

Reconfigurable antennas can operate in different frequency bands selectively by the employment of switching technology. The shortcoming of non-simultaneous operating in different frequency bands can be overcame by fixed multi-band antennas. However, fixed operating frequency bands of the antenna need to cover precisely the multiple operating frequency bands of the multi-standard communication networks, which makes the antenna sensitive to frequency shift because of manufacturing errors, environment effects [4]. Comparatively, UWB antennas provide a more competitive and simpler method for simultaneously operating in multi-standard communication networks with a small space. A uniform radiation pattern over the operating frequency band and stable gain against frequency are important parameters of a UWB antenna. Worse signal performance of a UWB antenna will be generated because of the dispersion caused by a varied radiation pattern and the fluctuating gain [5].

Numerous papers have been published for the design of UWB antennas. Frequency independent antennas (such as spiral antennas [6,7,8,9] and log-periodic antennas [10,11,12]) were developed for operating in ultra-wideband. However, due to phase center floating with frequency, dispersion may be generated in the frequency independent antennas. Horn antennas and reflector antennas were employed in ultra-wideband regardless of their large bulk [13,14,15,16]. Electronic-magnetic structured antennas were proposed in [17,18,19,20]. The antennas had a quarter-wave profile height employed in UWB applications. A Vivaldi antenna was introduced in UWB applications as well [21]. However, the antenna exhibited end fire radiation over the ultra-wideband. Besides, the profile of the antenna was large (one wavelength at the lowest frequency) in the radiated direction. Miniaturization was obtained by introducing an antipodal structure fed directly by a microstrip feeder [22]. Corrugated structures were also utilized in the radiator and the ground plane etched on the substrate (Rogers RT6010, ԑ_r_ = 10.2, Rogers company, Chandler, Arizona, U.S.) of high dielectric constant to achieve low profile but at higher costs than FR4 substrate. The low profile and side fire radiation can be achieved by planar monopole antennas [4,23,24,25] and wide slot antennas [26,27,28,29,30]. The planar monopole antennas [23,24,25] had large dimensions which are larger than the half of the wavelength. A miniaturized fork shape antenna was presented in [4] that obtained the gain of 2.38–11.8 dBi over the frequency band of 2.38–11.8 GHz, while the structure was relatively complicated. Qing [27] presented a monopole-like slot antenna with the gain of 1–4 dBi over the band of 2.7–12.4 GHz. A similar structure was proposed with the gain of 1–5.5 dB over the band of 3.1–11.1 GHz [30]. To improve the gain in the low frequency band, a rectangular slot antenna was proposed with the gain of 2–4.5 dBi over the band of 3.04–10.87 GHz [26]. Similarly, Li [28] presented the elliptical/circular slot antennas with the gain of 2–7 dBi over the band of 3.1–10.6 GHz in a larger size. However, according to those papers, gain decreases with the decrease of frequency, especially in the low frequency band. It is challenging to achieve a high gain in low frequency band. We present a novel antenna structure with a smaller size than the antennas presented [23,24,25,28,29]. The proposed antenna can achieve enhanced stable gain of 2.35–4.85 dBi over the band of 1.7–6.3 GHz. A higher gain at a lower frequency and better signal performance can be obtained. The proposed antenna may be utilized with the help of smart selective networks for the applications of 3G, 4G, 5G (sub 6 GHz), WIFI, and Bluetooth. Furthermore, in recent years, the underutilization of the frequency has inspired the generation of the cognitive radio (CR). A CR is capable of sensing the spectrum and changing system parameters, such as the frequency, transmitted power, or standard, if required. A UWB antenna is used for spectrum sensing whilst a reconfigurable narrowband antenna is employed for communication [31,32]. Therefore, the proposed antenna can be employed for spectrum sensing. As for the reconfigurable antenna, the proposed antenna can integrate easily with the smart tuning circuits for a reconfigurable radio application [33].

In this paper, a novel low-profile UWB boat-radiator antenna (BRA) is demonstrated. The antenna is composed of a boat-radiator and a dual C-shape co-radiative ground (DCCRG). One half of the DCCRG plays a role of the ground of a co-planar waveguide fed to the proposed boat-radiator antenna, while the other half works as a multiple order L-resonant circuit to broaden the lower operating band. The proposed antenna exhibits uniform bidirectional radiation characteristics with the size of 0.25 λ × 0.375 λ × 0.0063 λ over the frequency band of 1.7–6.3 GHz (115%). The proposed antenna achieved around twice the bandwidth (60%) of the antenna with the same structure without the structure acting as a multiple order L-resonant circuit. Moreover, the stable boresight gain of 3.6 dBi ± 1.25 dBi is realized over the operating band. The proposed antenna can be applied as a multiple functional antenna operating for multi-standard communication networks without the interference among antennas, and it is low-cost and simply fabricated.

## 2. Configuration and Principle of Proposed BRA

**Figure 1:**
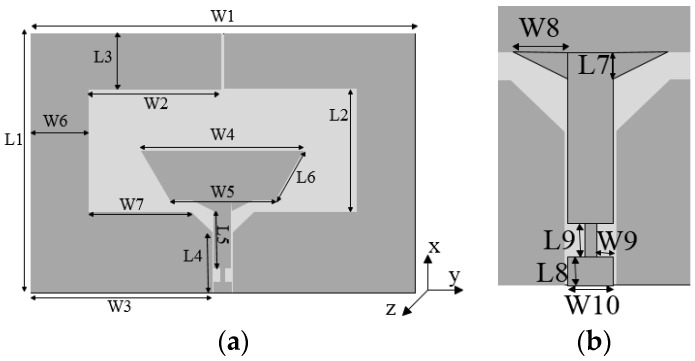
Geometry of proposed BRA: (**a**) top view of BRA; (**b**) the view of the high impedance stub (HIS).

The geometry of the BRA is shown in Figure 1 with detailed dimensions in Table 1. The antenna consists of a boat-radiator and a dual C-shape co-radiative ground (DCCRG). The boat shape is a half of the hexagon. It is chosen as a radiator for its good matching characteristics with wave impedance in free space. By combining the boat-shape and the chamfer in the feed line, gradual impedance is realized to achieve good matching. In addition, miniaturization in *x*-axis direction can be achieve using the boat-radiator instead of the hexagon. Two C-shape structures are placed symmetrically around the boat-radiator. All of them are copper plane printed on the top of a 1.1-mm-thick substrate (FR4) with a relative dielectric constant of 4.4 and loss tangent of 0.02. The DCCRG can be divided into two parts along the *y*-axis direction. One half of the DCCRG plays the role of the ground of a co-planar waveguide fed to the proposed boat-radiator antenna, while the other half is composed of two inverted aspectant L-shape structures that work as a multiple order L-resonant circuit to broaden the lower operating band. Meanwhile, the series high-impedance stub (HIS) is employed in the feed line, which is an effective way to adjust the input impedance to 50 Ω matched with the characteristic impedance of small A type (SMA) interface (50 Ω). The theoretical characteristic impedance of the high-impedance stub is 104 Ω in the proposed antenna. The overall size of the proposed design is 0.375 λ × 0.25 λ × 0.0063 λ.

**Figure 2:**
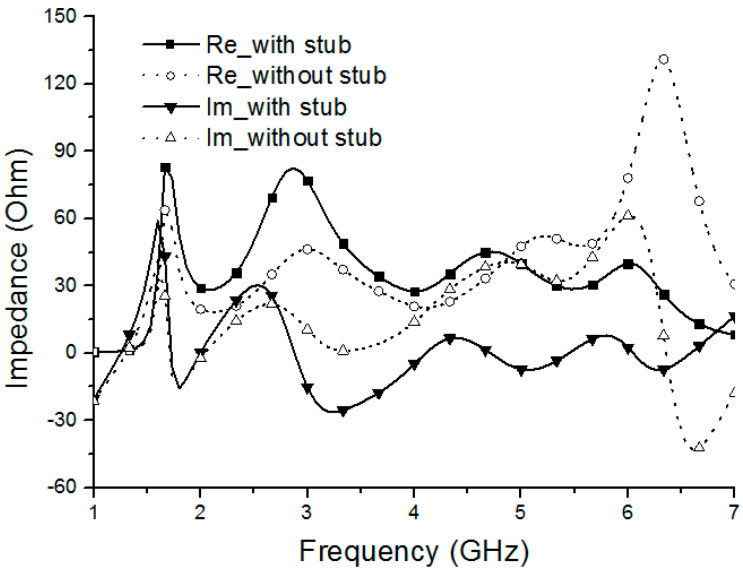
Comparison of the input impedance between the antenna with and without the high-impedance stub (i.e., with stub and without stub).

**Figure 3:**
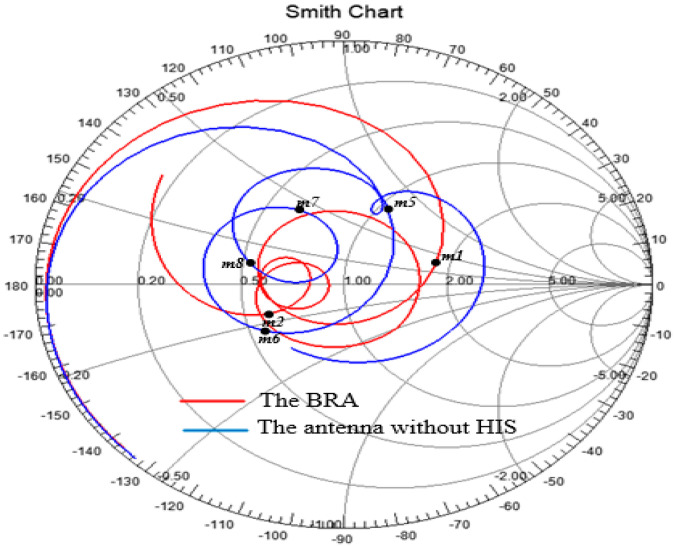
Comparison of input impedance characteristics between the proposed BRA and the antenna without HIS by Smith Chart (marker point: m1, 1.7 GHz; m2, 6.3 GHz; m5, 1.65 GHz; m6, 1.85 GHz; m7, 2.64 GHz; m8, 3.71 GHz).

To study the performance of the HIS, Figure 2 illustrates the effect of high-impedance stub (HIS) on the input impedance of the antenna compared with the same structure without HIS. The data were obtained by the finite element method (FEM). It can be observed that the real part of the input impedance of the antenna (BRA) with HIS is improved over the main operating band compared with that of the antenna without HIS. Therefore, the real part of the input impedance can be adjusted to around 50 Ω. Similarly, the imaginary part of the input impedance of the BRA is decreased over the main operating band compared with that of the antenna without HIS, which means that the imaginary part of the input impedance of the BRA is adjusted to about 0 Ω. Thus, proposed BRA can be matched with SMA connector (50 Ω) over the wideband of 1.7–6.3 GHz. Moreover, the input impedance characteristics of the proposed BRA presented by Smith chart are depicted in Figure 3 in comparison with those of the antenna without HIS. The marker points m1 (1.7 GHz) and m2 (6.3 GHz) are the starting frequency point and ending frequency point of the operating band (|S11| < −10 dB) of the BRA, respectively. Similarly, the marker points m5 (1.65 GHz), m6 (1.85 GHz), m7 (2.64 GHz), and m8 (3.71 GHz) are the starting frequency point and ending frequency point of the main operating bands (|S11| < −10 dB; 1.65 GHz–1.85 GHz, 2.64 GHz–3.71 GHz) of the antenna without HIS, respectively. It can be observed that the curve of BRA between m1 and m2 is tight around the normalized 1 Ω point, which means good matching characteristics can be obtained in that frequency band. However, only the curve between m5 and m6 and the curve between m7 and m8 with respect to the antenna without HIS are tightly around the normalized 1 Ω point, which means that good matching characteristics can be obtained in those frequency bands. Therefore, enhanced matching characteristics can be obtained by employing the HIS.

**Figure 4:**
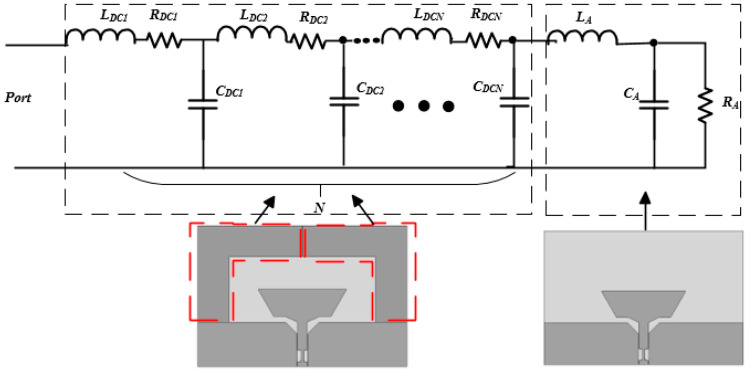
The equivalent circuit of the proposed BRA.

Furthermore, the performance of the DCCRG is studied. As shown in Figure 1a, DCCRG is fabricated around the boat-radiator. The half of the DCCRG plays a role of the ground of a co-planar waveguide fed to the proposed boat-radiator antenna, while the other half works as a multiple order L-resonant circuit to broaden the lower operating band. As we know that the co-planar waveguide as a feed line is helpful for wideband characteristics [22], in this paper, the performance of the other half of DCCRG is considered. The proposed BRA can be indicated as an equivalent circuit, as shown in Figure 4.

As shown in Figure 4, *L_A_*, *R_A_*, and *C_A_* represent the inductance, resistance, and capacitance of the antenna without the upper half of DCCRG (the area covered with red dotted lines), respectively. Furthermore, the *N* order L-resonant circuit is introduced as the equivalent circuit of the upper half of DCCRG (the area covered with red dotted lines). A series of *C_DCN_*, *L_DCN_*, and *R_DCN_* represent the capacitance, inductance, and resistance used in the *N* order L-resonant circuit, respectively.

**Figure 5:**
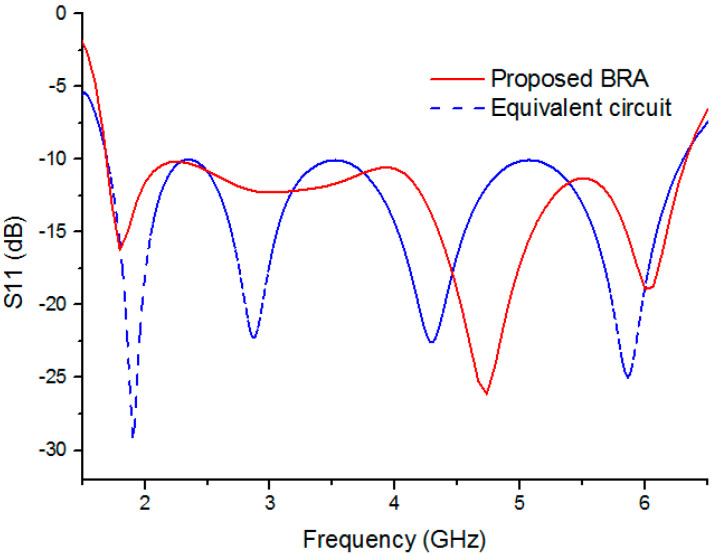
Comparison of the S11 between the BRA and the equivalent circuit.

To obtain more accurate parameter values of the *N* order L-resonant circuit, we extracted the input impedance matrix of the antenna without the upper half of DCCRG solved by the FEM solver. Then 5 (*N* = 5) order L-resonant circuit was cascaded with that input impedance matrix according to the equivalent circuit shown in Figure 4. The parameter values of the 5-order L-resonant circuit can be solved by the software Ansoft designer (Ansys company, Pittsburgh, Pennsylvania, USA) as shown in Table 2. Figure 5 depicts the reflection coefficient (S11) versus frequency of the equivalent circuit of the BRA compared with that of the proposed BRA. Due to the challenging nature of the perfect replacement between lumped components of the 5-order L-resonant circuit and distributed comments of the upper half of DCCRG, drastic fluctuation may be generated in the performance of the equivalent circuit. Moreover, complex coupling characteristics between the DCCRG and the boat-radiator are ignored to simplify the equivalent circuit. Therefore, the deviation may be generated between the performance of the equivalent circuit and the BRA. With the help of the 5-order L-resonant circuit, the operating band of the antenna without the upper half of DCCRG is expanded to lower frequency (1.7 GHz) which is the same as the operating band of the proposed BRA. Moreover, the same variation trend between two curves is realized. Therefore, S11 of the equivalent circuit is consistent with that of the BRA within acceptable error. The equivalent circuit can be used to study impedance characteristics of the proposed BRA within acceptable error.

**Figure 6:**
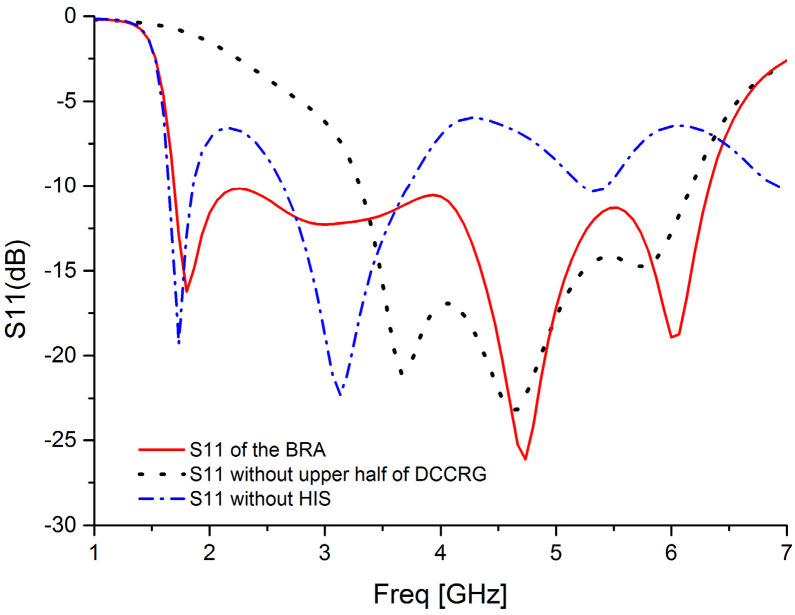
Comparison of the S11 between the BRA (**the red line**) and the antenna without upper half of DCCRG (**the black line**), and S11 of the antenna without HIS (**the blue line**).

**Figure 7:**
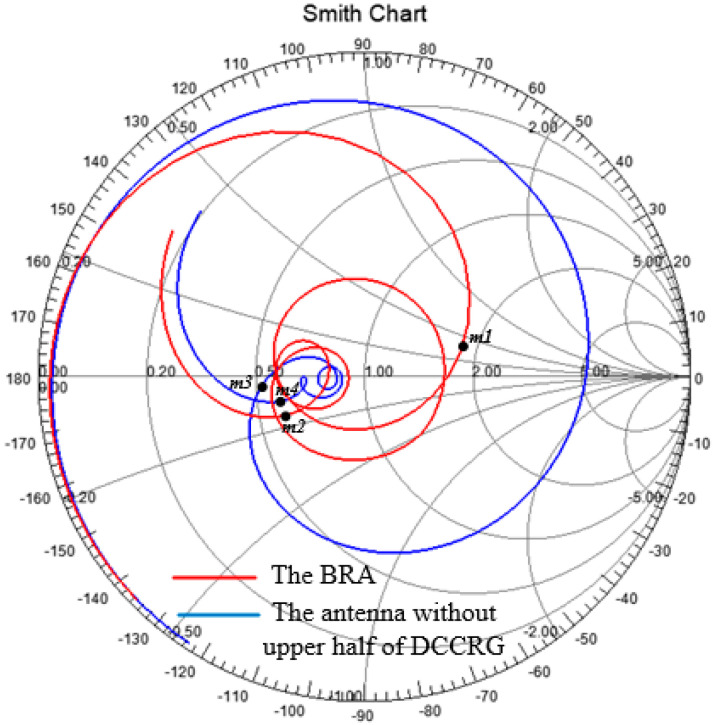
Comparison of input impedance characteristics between the proposed BRA and the antenna without upper half of DCCRG by Smith Chart (marker point: m1, 1.7 GHz; m2, 6.3 GHz; m3, 3.3 GHz; m4, 6.1 GHz).

To reveal the performance of DCCRG, Figure 6 depicts the reflection coefficient (S11) against frequency of the proposed BRA (the red line) compared with the antenna without the upper half of the DCCRG (the black line). Moreover, S11 of the antenna without the high-impedance stub (HIS, the blue line) is also shown in Figure 6. Those simulated data were also obtained by FEM. It can be seen that the proposed BRA can achieve the operating frequency band of 1.7–6.3 GHz (115%). The band is around twice the operating band (3.3–6.1 GHz, 60%) of the same structured antenna without the upper half of DCCRG. In addition, the input impedance characteristics of the proposed BRA presented by the Smith chart is depicted in Figure 7 compared with that of the antenna without the upper half of the DCCRG. As mentioned above, the marker points m1 (1.7 GHz) and m2 (6.3 GHz) are the starting frequency point and ending frequency point of the operating band (|S11| < −10dB) of the BRA, respectively. Similarly, the marker points m3 (3.3 GHz) and m4 (6.1 GHz) are the starting frequency point and ending frequency point of the operating bands (|S11| < −10dB) of the antenna without the upper half of the DCCRG, respectively. It can be observed that the curve of BRA between m1 and m2 is tight around the normalized 1 Ω point, which means good matching characteristics can be obtained in that frequency band. However, only the curve between m3 and m4 with respect to the antenna without upper half of DCCRG is tightly around the normalized 1 Ω point, which means that good matching characteristics can be obtained in that frequency band. Therefore, it is helpful for achieving lower operating frequency and broadening bandwidth to employ the structure of DCCRG.

Moreover, since the input impedance matching characteristics of the antenna without the HIS is worse than that of the proposed BRA (see Figure 2), S11 of the proposed BRA is lower than S11 of the antenna without HIS over the most of operating frequency band. Therefore, a better performance of matching can be achieved by employing HIS.

**Figure 8:**
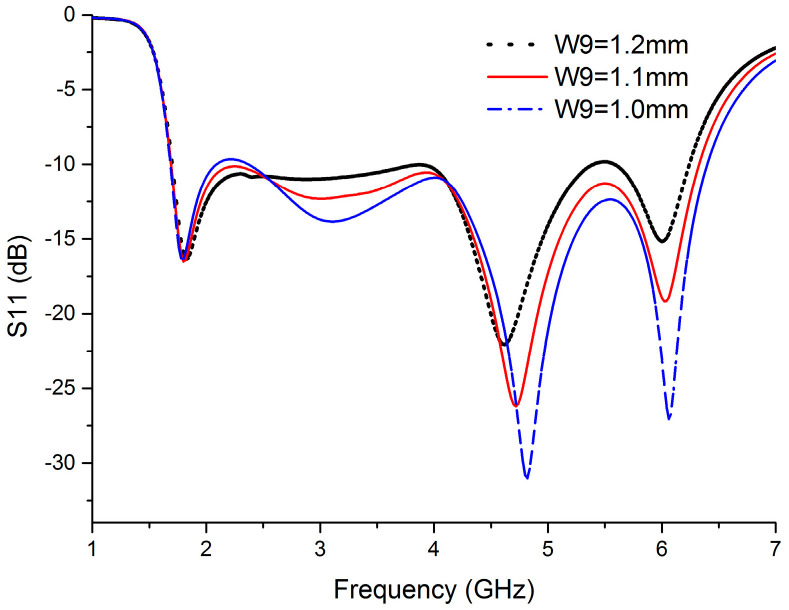
Reflection coefficient (S11) of the BRA versus frequency with the variation of W9.

**Figure 9:**
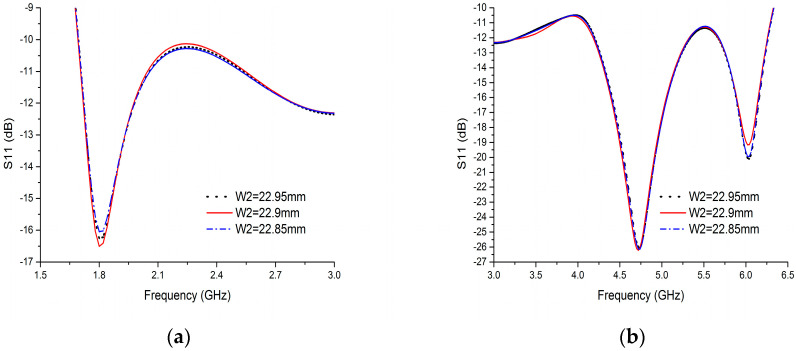
Reflection coefficient (S11) of the BRA versus frequency with the variation of W2: (**a**) 1.5 GHz–3 GHz; (**b**) 3 GHz–6.5 GHz.

The width of HIS is the key parameter which affects the performance of HIS. It is determined by the parameter of W9. Therefore, the effect of variation in W9 value on reflection coefficient (S11) of the proposed BRA is simulated in Figure 8. With the increase of W9 value, the width of HIS is decreased. The S11 in the frequency band of 1.8–2.5 GHz decreases, while the S11 in the most of frequency band of 2.5–6.3 GHz increases. Thus, the optimized point of W9 is 1.1mm. The performance of the structure of upper half of DCCRG is affected by the width of the slot between the left upper half of DCCRG and the right upper half of DCCRG which is determined by W2 value. Therefore, the effect of variation in W2 value on reflection coefficient (S11) of the proposed BRA is simulated in Figure 9. It can be seen that S11 is slightly varied with the variation in W2 value. When W2 = 22.9 mm, S11 is lower than other curves of S11 in most of the operating bands except the frequency bands of 2–2.8 GHz and 5.8–6.1 GHz. Therefore, the optimized point of W2 is 22.9 mm.

## 3. Performance of Proposed BRA

**Figure 10:**
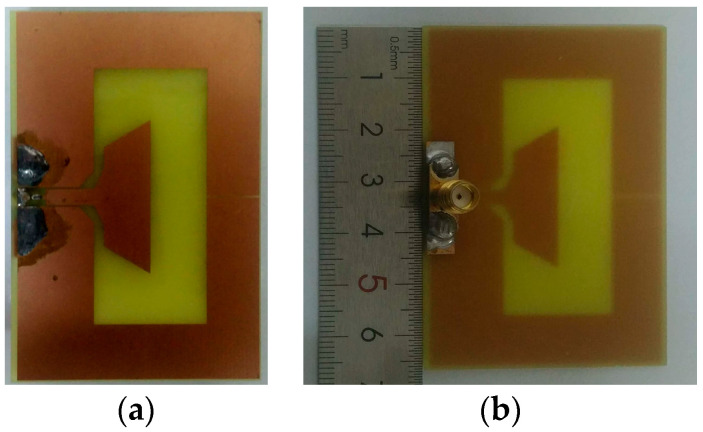
Prototype of the proposed BRA: (**a**) front view; (**b**) back view.

To validate operating bandwidth and the radiation performance of the proposed BRA, the presented antenna was numerically optimized by finite element method (FEM) solver and a prototype (see Figure 10) of the antenna was fabricated to measure. The measurements were carried out by the usage of vector network analyzer Angilent N5245A (Keysight company, Santa Rosa, CA, USA) in the anechoic chamber.

**Figure 11:**
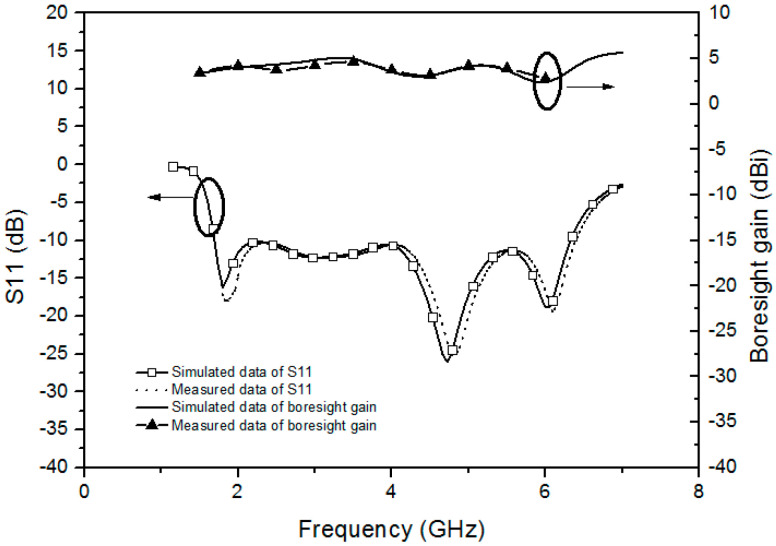
The measured S11 and boresight gain of the proposed BRA compared with simulated data.

Figure 11 shows the simulated and measured reflection coefficient (S11) and the boresight gain of the proposed antenna. Regarding this figure, the measured impedance matching bandwidth is in good agreement with the simulated impedance matching bandwidth within acceptable tolerance. Within the operating band, the measured boresight gain is 3.6 dBi ± 1.25 dBi consistent with the simulated data within little tolerance.

**Figure 12:**
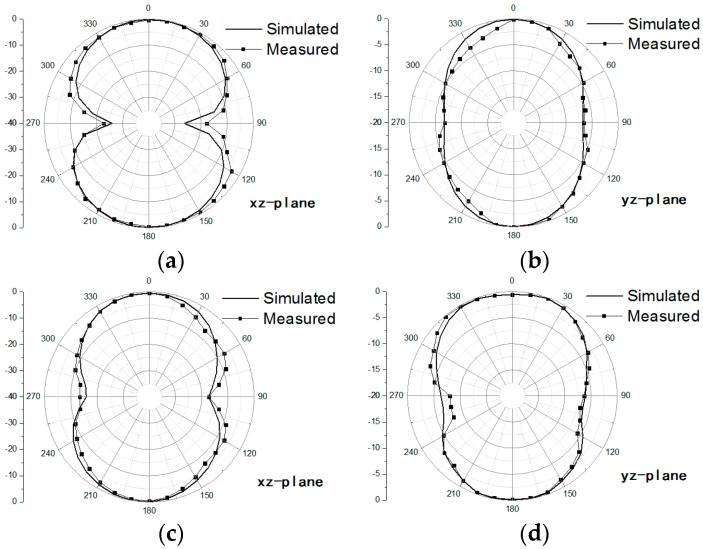

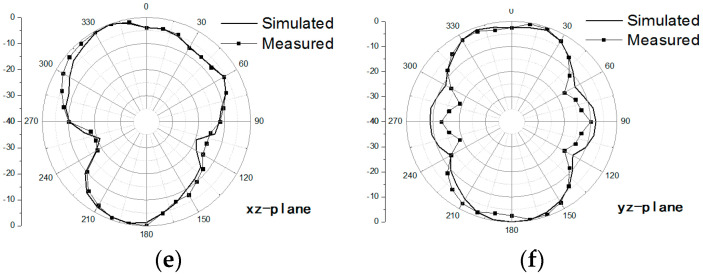
The Simulated and measured gain pattern of the proposed antenna in normalization at 2 GHz (**a**,**b**), 4 GHz (**c**,**d**) and 6 GHz (**e**,**f**).

The simulated and measured gain patterns at frequencies of 2, 4, and 6 GHz, respectively, are shown in Figure 12. Notice that these patterns are normalized with their maximum gain. The structures of the DCCRG and boat-radiator can be considered as a monopole array with three monopole elements oriented in *y*-axis, which causes two nulls along *y*-axis in *yz*-plane. The radiation along the *y*-direction is degraded because of the array factor whereas the radiation in the *z*-direction is still kept maximal. While two nulls along *x*-axis in *xz*-plane are generated because of radiation characteristics of a monopole antenna. Due to asymmetric structure of proposed BRA along the *x*-direction, slight squinting of the radiation pattern in the *xz*-plane is observed at 6 GHz. It can be observed that the simulated gain patterns are in good agreement with the measured data. Within the coverage of the operating frequency band, the bidirectional radiation patterns are almost consistent whether in the *xz*-plane or in the *yz*-plane. Bidirectional radiation patterns at frequencies of 2 GHz and 4 GHz can demonstrate great consistency. Moreover, the radiation pattern at the frequency of 6 GHz is in good agreement with the one at the frequency of 4 GHz within acceptable tolerance. Therefore, stable radiation patterns over the operating frequency band can be achieved for the proposed BRA.

Group delay is an important parameter for UWB communications since it can be used to judge the waveform distortion in the time domain. In the experiment, two identical reference antennas were placed face to face over a distance of *r* = 40 cm to meet far-field region condition, given by:

(1) $$r=\frac{2L^{2}}{\lambda v}$$

where *L* = 80 mm and is the diameter of a sphere which contains the structure of the antenna and *λ*_v_= 42.8 mm is the wavelength in free space corresponding to higher operating frequency.

**Figure 13:**
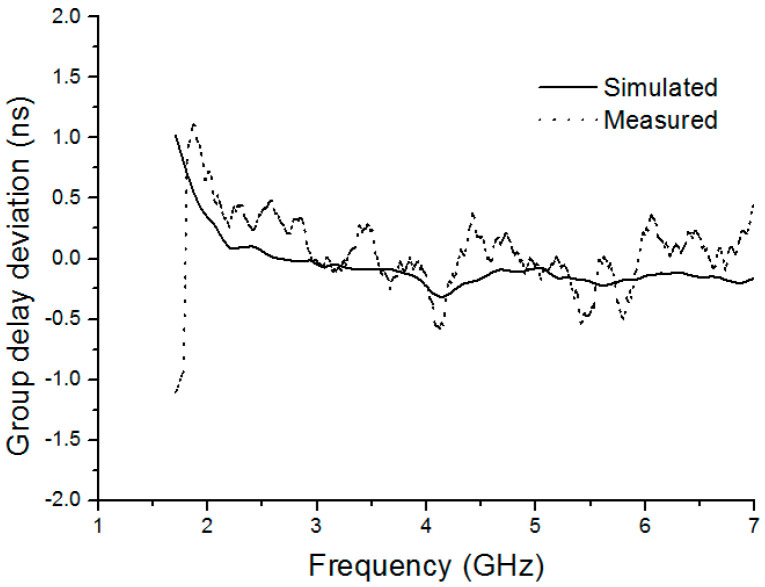
Simulated and measured group delay deviation of the proposed BRA.

One antenna is as a TX antenna to transmit signal, and the other one is as an RX antenna to receive it. For a perfect pulse transmission, the group delay should be almost constant within the entire operating band. Therefore, group delay deviation denoted as the difference between the group delay and the mean of the group delay over the operating band should be zero against frequency. Both simulated and measured data in Figure 13 illustrate that the group delay deviation is less than ±1.12 ns over the operating band. The trend of the measured data is consistent with the trend of the simulated data. The group delay deviation tends to zero in the operating frequency band greater than 2 GHz. Therefore, a flat group delay has been achieved with tolerance, which means the proposed UWB antenna has a good linear phase response.

Table 3 compares the proposed BRA and the antennas available in literature [4,23,24,25,26,27,28,29,30]. All those antennas have the planar monopole structure or the wide slot antenna structure. It can be seen that the proposed antenna realizes smaller dimensions than the antennas proposed in literature [23,24,25,28,29]. As mentioned above, stable uniform radiation over the operating frequency band can realize great signal performance due to low dispersion [5]. Furthermore, according to those references, gain decreases with the decrease of frequency, especially in the low frequency band. It is challenging to achieve high gain in the low frequency band. However, the proposed antenna realizes the higher gain at a lower frequency to maintain enhanced gain stability compared with antennas presented in [4,26,27,30]. Better gain performance of the proposed antenna makes the design of smart tuning networks easier without the power compensation in the future. The proposed antenna can be used with the help of the smart tuning networks for a micro-site or a pico-site base station instead of the directional radiated antenna to decrease the numbers of antennas [34]. Furthermore, the proposed antenna can be employed for spectrum sensing of CR applications. As for the reconfigurable antenna for CR, the smart tuning circuits can integrate easily with the proposed antenna for the reconfigurable application [33]. In the future, a smart tuning circuit will be studied to achieve intelligent frequency section flexibly and precisely [35].

## 4. Conclusions

In this paper, a novel low-profile UWB boat-radiator antenna (BRA) has been demonstrated. It is composed of a boat-radiator and a dual C-shape co-radiative ground (DCCRG). One half of the DCCRG plays a role of the ground of a co-planar waveguide fed to the proposed boat-radiator antenna, while the other half works as a multiple order L-resonant circuit to broaden the lower operating band. The equivalent circuit of the proposed antenna has been presented. The proposed antenna exhibits uniform bidirectional radiation with the size of 0.25 λ × 0.375 λ × 0.0063 λ over the frequency band of 1.7–6.3 GHz (115%). The operating band is around twice the bandwidth (60%) of the same structured antenna without the structure acting as a multiple order L-resonant circuit. Moreover, the stable boresight gain of 3.6 dBi ± 1.25 dBi over the operating band has been achieved by simulations and measurements. Furthermore, a flat group delay has been achieved with tolerance, which means that the proposed antenna has a good linear phase response. This antenna can operate for multi-standard communication networks, including cognitive radio applications without interference, and it is low-cost and simply fabricated.

## Figures and Tables

**Table 1:** Dimensions of proposed BRA.

Parameters	Value (mm)	Parameters	Value (mm)
L1	44.4	L2	20.7
L3	9.7	L4	11.2
L5	10.6	L6	9.92
L7	1.8	L8	1.6
L9	3.6	W1	66
W2	22.9	W3	31.28
W4	27.92	W5	18
W6	10	W7	28.48
W8	3.7	W9	1.1
W10	3		

**Table 2:** Parameters of 5 order L-resonant circuit.

Parameters	Value	Parameters	Value
*L_DC_*_1_	0.39 nH	*L_DC_*_2_	1.13 nH
*L_DC_*_3_	0.87 nH	*L_DC_*_4_	0.74 nH
*L_DC_*_5_	0.68 nH	*C_DC_*_1_	1.14 pF
*C_DC_*_2_	2.36 pF	*C_DC_*_3_	3.94 pF
*C_DC_*_4_	9.97 pF	*C_DC_*_5_	11.82 pF
*R_DC_*_1_	2.43 Ω	*R_DC_*_2_	2.51 Ω
*R_DC_*_3_	2.03 Ω	*R_DC_*_4_	2.18 Ω
*R_DC_*_5_	1.9 Ω		

**Table 3:** Comparison of the proposed antenna with the antennas available in literature.

Literature	Dimensions (λ^3^)	Bandwidth (GHz)	Gain (dBi)	Radiation Pattern	Applications
[4]	0.22 × 0.25 × 0.013	2.38–11.8 (132%)	2.3–5.6	bidirectional	WLAN/LTE/UWB
[23]	0.85 × 0.65 × 0.048	3.18–11 (110%)	1.1–7.2	directional	WBAN
[24]	0.54 × 0.65 × 0.0085	3.2–10.9 (109%)	1.2–5.1	bidirectional	GSM/PCS/WLAN
[25]	0.68 × 0.75 × 0.0044	2.6–11 (123%)	1.3–5.3	bidirectional	WIMAX/UWB
[26]	0.21 × 0.21 × 0.0082	3.04–10.87 (112%)	2–4.5	bidirectional	UWB
[27]	0.24 × 0.27 × 0.014	2.7–12.4 (128%)	−1–4	bidirectional	UWB
[28]	0.41 × 0.39 × 0.016	3.1–10.6 (109%)	2–7	bidirectional	UWB
[29]	0.44 × 0.44 × 0.0037	2.2–30 (172%)	- ^1^	bidirectional	UWB
[30]	0.27 × 0.27 × 0.017	3.1–11.1 (112%)	−1–5.5	bidirectional	UWB
proposed	0.25 × 0.3 × 0.0063	1.7–6.3 (115%)	2.35–4.85	bidirectional	3G/4G/5G/WIFI/Bluetooth

^1^ This data is unavailable in literature.
